# *N,N*-Dimethyl Formamide Regulating Fluorescence of MXene Quantum Dots for the Sensitive Determination of Fe^3+^

**DOI:** 10.1186/s11671-021-03617-9

**Published:** 2021-10-28

**Authors:** Xiaohui Gao, Xiaochun Shao, Longlong Qin, Yejun Li, Shengxiang Huang, Lianwen Deng

**Affiliations:** School of Physics and Electronics, Central South University, Changsha, 410083 China

**Keywords:** MXene quantum dots, Fluorescence, Detection of Fe^3+^

## Abstract

Due to the wide use of iron in all kinds of areas, the design and construction of direct, fast, and highly sensitive sensor for Fe^3+^ are highly desirable and important. In the present work, a kind of fluorescent MXene quantum dots (MQDs) was synthesized via an intermittent ultrasound process using *N,N*-dimethyl formamide as solvent. The prepared MQDs were characterized via a combination of UV–Vis absorption, fluorescence spectra, X-ray photoelectron energy spectra, and Fourier-transform infrared spectroscopy. Based on the electrostatic-induced aggregation quenching mechanism, the fluorescent MQDs probes exhibited excellent sensing performance for the detection of Fe^3+^, with a sensitivity of 0.6377 mM^−1^ and the detection limit of 1.4 μM, superior to those reported in studies. The present MQDs-based probes demonstrate the potential promising applications as the sensing device of Fe^3+^.

## Introduction

MXene quantum dots (MQDs), originating from 2D transition metal carbides or nitrides, show appealing physical and chemical properties including abundant metal-deficient sites, excellent charge or electrons transport ability, and good biocompatibility, which greatly contribute to the wide range of applications in energy storage, catalysis, sensors, thermoelectricity, and bio-imaging [1–5]. In recent years, because of the appropriate band gap, easy surface modification, and the quantum size effect, the fluorescent properties of MQDs are gradually emerging as a great application prospect in the optical-sensing field such as the detection of metal ions, hypochlorite, glutathione, and hypochlorite [6–8]. As reported, the performance of quantum dots-based sensors substantially depends on the optical and surface/interfacial properties of materials, especial for the MQDs [9–11]. Meanwhile, considerable research efforts have been devoted into the synthesis of MQDs and understanding the critical roles of the surface capping organic ligands and the solvents used in the synthesis process. For example, Zhou et al. synthesized the nitrogen-doped Ti_3_C_2_ QDs combined with *2,3-*diaminophenazine, presenting a sensitively ratiometric sensor for H_2_O_2_ and xanthine. The limit of detection was determined to be 0.57 and 0.34 μM, respectively [12]. By integrating the electron transfer and inner filter effect, Liu et al. reported the fluorescent MQDs synthesized in dimethyl sulfoxide (DMSO) for the detection of Fe^3+^ with high sensitivity and selectivity [13]. Despite of these, the current studies about fluorescent MQDs-based sensors are still limited, especially for metal ions, and the constructions of corresponding devices have not yet been developed. Meanwhile, the exploration of the relationship between optical and interfacial properties of MQDs is still in infancy.

Iron, as an indispensable metal, has been widely used in all kinds of areas. On the one hand, large quantities of wastewater containing ferric ions are constantly released to the natural environment, which is detrimental to the microorganism and the food chain [14–16]. On the other hand, the level of iron ions in blood is critical to the health of human body, and the corresponding disorder can cause the serious physiological responses, including cardiopalmus, anemia, and the dysfunction of organs [17, 18]. Therefore, the accurate determination of iron content is of great importance to the sustainable development of mankind and society. To date, all kinds of analytical techniques have been utilized to the detection of Fe^3+^, including atomic absorption spectrometry, inductively coupled plasma mass spectrometry, colorimetry, and electrochemistry [19–21]. Among these methods, fluorometric analysis offers some unique advantages such as high sensitivity, rapid response, and good selectivity. Various fluorescent nanomaterials have also been developed for the analysis of Fe^3+^, e.g., quantum dots, small molecule probes, metal–organic frameworks, and metal nanoclusters [22–26]. However, it is worthy to be mentioned that the existing sensitivity and selectivity remain significant challenges for in situ and portable detection. The research and development of direct, fast, and highly sensitive probes for Fe^3+^ are still desirable and important.

Therefore, in this work, a kind of fluorescent MQDs was synthesized via an intermittent ultrasound process with *N,N*-dimethyl formamide as solvent. The prepared MQDs were characterized by UV–Vis absorption, fluorescence spectra, X-ray photoelectron energy spectra, and Fourier-transform infrared spectroscopy. Based on the electrostatic-induced aggregation quenching mechanism, the fluorescent MQDs probes exhibited the excellent sensing performance for the detection of Fe^3+^. The sensitivity was determined to be 0.6377 mM^−1^ with the detection limit of 1.4 μM, superior to those reported in studies. We believe that the present MQDs-based probes will be a promising candidate for the sensing device of Fe^3+^.

## Methods and Experiments

### Chemicals and Materials

Bulk titanium aluminum carbide powders (Ti_3_AlC_2_, 98%) were purchased from Beijing Forsman Scientific Co., Ltd. Hydrofluoric acid (HF, A.R., ≥ 40%), zinc nitrate hexahydrate (Zn(NO_3_)_2_^.^6H_2_O, A.R.), sodium chloride (NaCl, A.R.), and potassium chloride (KCl, A.R.) were brought from Sinopharm Chemical Reagent Co., Ltd. (Shanghai). Iron nitrate nonahydrate (Fe(NO_3_)_3_^.^9H_2_O, A.R.), nickel nitrate hexahydrate (Ni(NO_3_)_2_^.^6H_2_O, A.R.), and cobalt nitrate hexahydrate (Co(NO_3_)_2_^.^6H_2_O, A.R.) were obtained from Guangdong Guanghua Sci-Tech. Co., Ltd. *N,N*-dimethyl formamide (C_3_H_7_NO, DMF, A.R.) and cupric nitrate trihydrate (Cu(NO_3_)_2_^.^3H_2_O, A.R.) were obtained from Shanghai Macklin Biochemical Co., Ltd. Aluminum nitrate nonahydrate (Al(NO_3_)_3_^.^9H_2_O, A.R.) was from Aladdin. Nitric acid (HNO_3_, 65–68%) was obtained from Chengdu Chron Chemicals Co., Ltd. Ammonium chloride (NH_4_Cl, A.R.) and magnesium chloride hexahydrate (MgCl_2_^.^6H_2_O, A.R.) were brought from Shanghai Zhanyun Chemical Co., Ltd., and Xilong Chemical Co., Ltd., respectively.

### Characterizations

Transmission electron microscopy (TEM) images were collected on Titan G2 60–300 with an acceleration voltage of 300 kV. X-ray photoelectron spectroscopy (XPS) experiments were conducted on a AVG Thermo VG ESCALAB 250 spectrometer equipped with a Mg Kα anode. Fourier-transform infrared (FTIR) spectra were recorded on a BRUKE Vertex-70 FTIR spectrometer. UV–Vis spectra were obtained on a UV-3000PC spectrometer (Shanghai Mapada Instrumental Co., Ltd.). Zeta potentials were measured on a Zeta Sizer Nano ZS (Malvern Instruments, UK). Fluorescence spectra were recorded by using F-4600 fluorescence spectrophotometer (Hitachi, Tokyo, Japan).

### Synthesis of MXene quantum dots

In a typical process, 20 ml of hydrofluoric acid was added into a Teflon container with 2 g of bulk Ti_3_AlC_2_ powders. The mixture was allowed to constantly stirring at room temperature for 48 h. During this process, the aluminum layers were etched and the primary products were collected through centrifugation and washed with plenty of ultrapure water until neutral. Subsequently, the obtained solid substances were dispersed into 50 ml of DMF, and the dispersion was intermittently sonicated for another 48 h. The yellow supernatants were collected as the final products after centrifugation and stored for further use.

### Fluorescence Detection of Fe^3+^ Ions

In a typical detection, Fe(NO_3_)_3_ solutions were prepared by diluting the stock solution (10 mM) with aqueous nitric solution (10 mM). Different volumes of Fe^3+^ solution were mixed with 300 μL of the as-prepared MXene quantum dots solution, and the fluorescence curves were measured at room temperature after 60 s. To examine the selectivity of MXene quantum dots toward Fe^3+^, other metal ions with a concentration of 10 mM (Na^+^, K^+^, Ni^2+^, Cu^2+^, Co^2+^, Zn^2+^, Mg^2+^, Al^3+^, and NH_4_^+^) were also tested and the corresponding changes of fluorescence intensity were recorded.

## Results and Discussion

### Synthesis and Characterizations

**Fig. 1:**
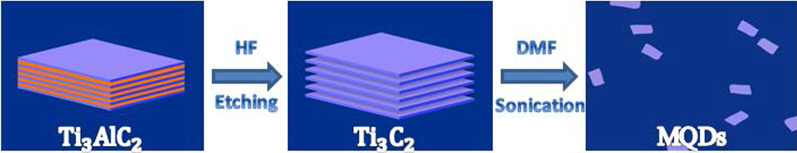
Schematic diagram for the preparation of MQDs

**Fig. 2:**
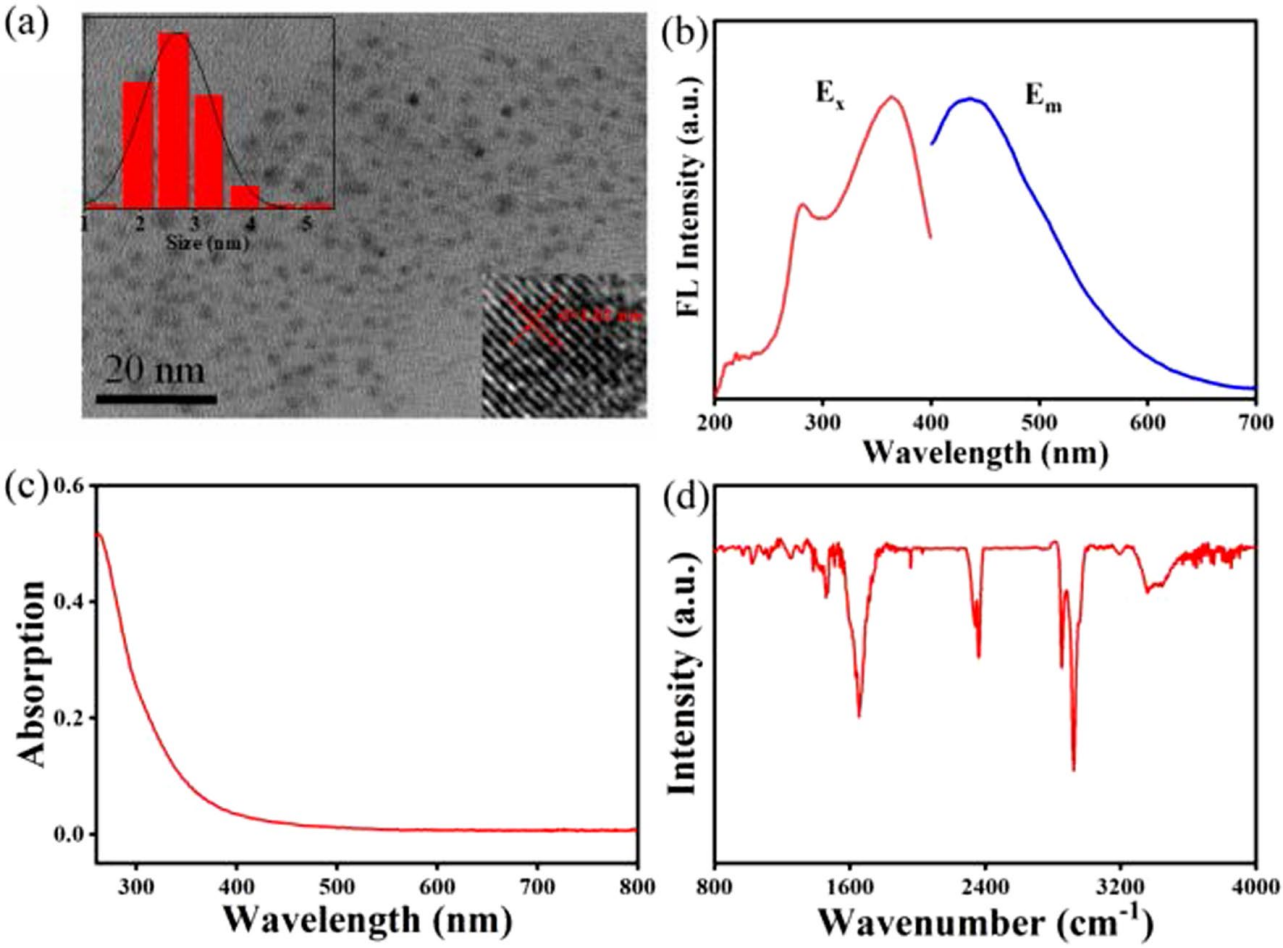
**a** TEM image of the synthesized MQDs with quasi-normal distribution of particle’s size image and the lattice spacing in the high-resolution image (left inset and low right inset); **b** fluorescence emission spectra of the prepared MQDs; **c** UV–Vis absorption spectrum; and **d** FTIR spectrum of MQDs

In this work, the synthesis of MQDs was completed through the intermittent sonication for 48 h. As shown in Fig. 1, by using hydrofluoric acid as etching reagent, the bulk Ti_3_AlC_4_ powders were first transformed into Ti_3_C_2_ nanosheets, which were subsequently cut into MQDs under the assistance of ultrasound and DMF solvent. To demonstrate the formation of MQDs, transmission electron microscopy (TEM) experiments were performed. As shown in Fig. 2a, in consistent with the previous reports, abundant MXene quantum dots instead of nanosheets were observed in the image [27–29]. Meanwhile, the low right inset in Fig. 2a displayed the high-resolution transmission electron microscopy image of MQDs. The lattice spacing was determined to be 1.02 nm, reasonably indicating the successful formation of MQDs. Based on a hundred particle count, the statistical average size of the obtained MQDs was estimated to be 2.75 nm, as shown in the left inset of Fig. 2a.

**Fig. 3:**
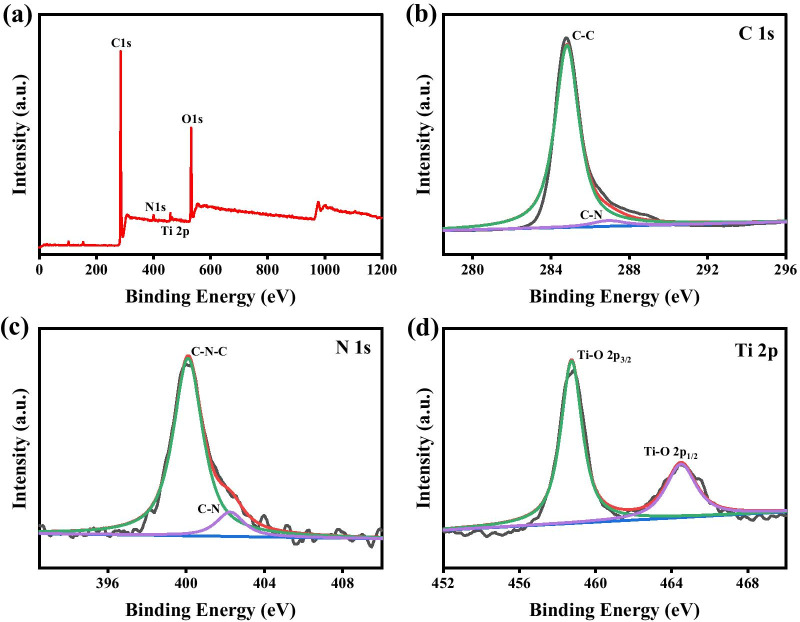
**a** XPS survey spectrum of MQDs; high resolution, **b** C 1*s*, **c** N 1*s*, and **d** Ti 2*p* XPS spectra of MQDs

As reported, the optical properties are one of the most fascinating part of quantum dots. In Fig. 2b, the fluorescence properties of the synthesized MQDs were revealed. The excitation and emission wavelength was determined at 365 and 445 nm, respectively, indicating the blue fluorescence at the excitation wavelength. Figure 2c shows the UV–Vis spectrum of MQDs. The absorption decreased with the wavelength increasing. The main absorption region was below 400 nm, indicating the high electronic energy level. By using quinine sulfate as a reference, the quantum yield of the MQDs was calculated to be 4.5%. To analyze the chemical bonds in the MQDs, Fourier-transform infrared spectroscopy (FTIR) experiments were conducted. As shown in Fig. 2d, the peaks at 1462 and 1654 cm^−1^ were originated from the stretching modes of C–N and C=O bond, respectively [30, 31]. The signals at 2894 and 2914 cm^−1^ were attributed to the C–H (–CH_3_ and –CH=O) stretching mode, indicating surface modification of the MQDs with the DMF molecules during the ultrasound process [32]. Note that the peak at 2365 cm^−1^ was resulted from the carbon dioxide in air. As well known, X-ray photoelectron spectroscopy (XPS) is sensitive to the chemical environment of the elements, which can be used to analyze the chemical valence states of elements. Figure 3a shows the survey spectrum from MQDs. As expected, the elements of Ti, C, O, and N were found in the prepared MQDs. The absence of aluminum signals indicates the complete etching of the intermediate layers. The resolved C 1*s* spectrum is presented in Fig. 3b. The C–C chemical bond was considered as the primary bonding mode on the basis of the relative high intensity. Due to the introduction of DMF, the C–N chemical bonds also existed in the prepared materials, which can be illustrated by the following N 1*s* spectrum. As shown in Fig. 3c, the signals of both C–N–C and C–N chemical bonds were presented in N 1*s* spectrum at the binding energy of 400.1 and 402.3 eV, respectively. For Ti 2*p* spectrum (Fig. 3d), the peaks at 458.7 and 464.3 eV were attributed to the Ti 2*p*_1/2_ and Ti 2*p*_3/2_ of Ti–O bonds, respectively, in consistent with the literature results [33, 34]. Therefore, combining with the TEM image, these results further illustrated the successful formation of MQDs with the modification of DMF molecules.

## The Sensitive and Selective Detection of Fe^3+^ Ions

**Fig. 4:**
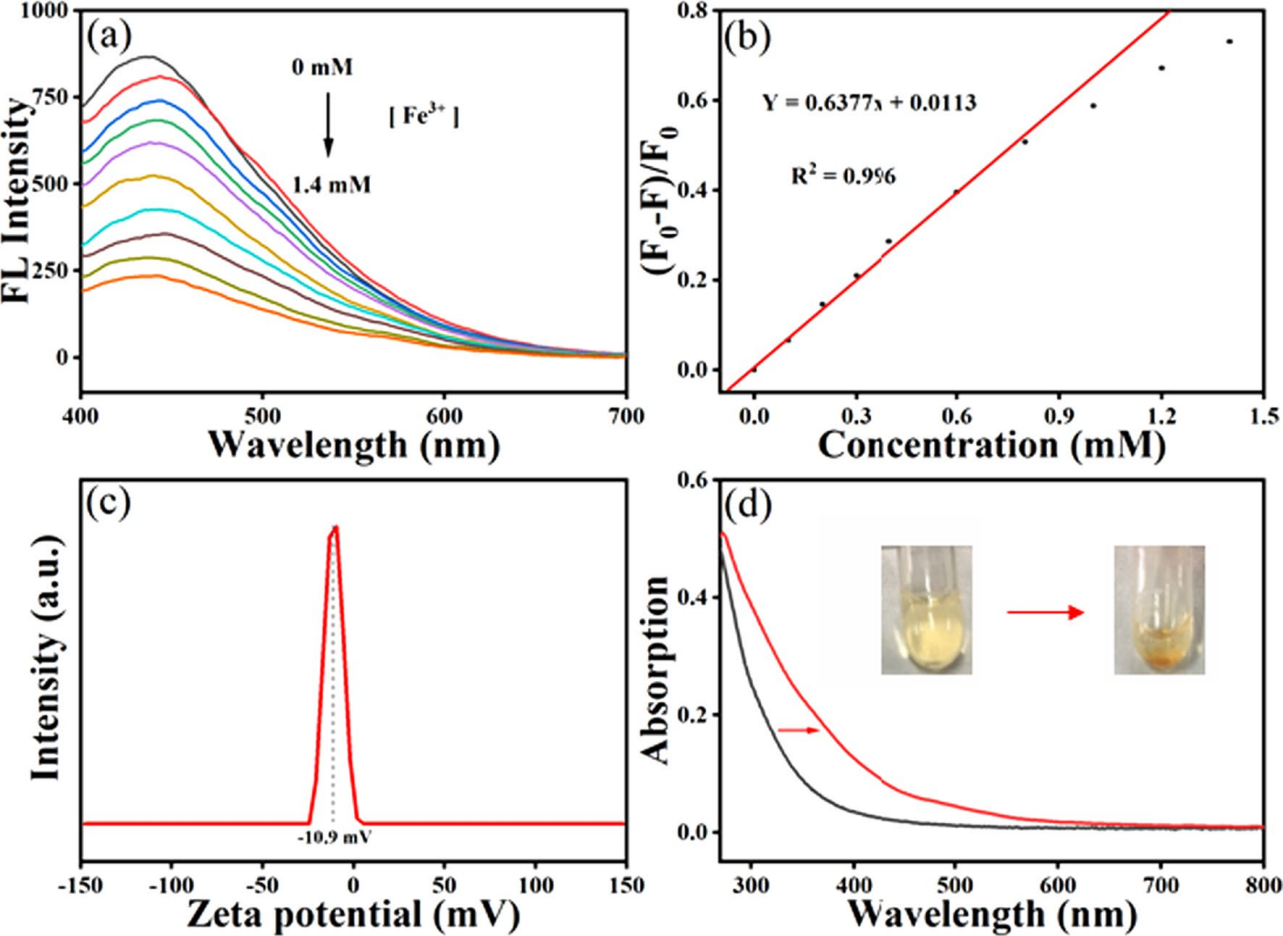
**a** Fluorescence emission spectra of MQDs with the addition of ferric ion; **b** calibration line between the concentration of Fe^3+^ and the fluorescence ratio; **c** the zeta potential of the prepared MQDs; and **d** UV–Vis absorption spectra of MQDs with and without the addition of Fe^3+^ions

Based on the high fluorescence intensity, the prepared MXene quantum dots were allowed to analyze the ferric ions in aqueous solution. As shown in Fig. 4a, the fluorescence intensity of MQDs was gradually decreasing with the addition of ferric ions, indicating an effective quenching effect. In particular, approximate 30% of fluorescence intensity was suppressed by the ferric ions with the concentration of 1.4 mM. From further quantitative investigation, the fluorescence response of the MQDs toward ferric ions with different concentrations was also examined. In Fig. 3a, b, linear relationship between (*F*_0_ − *F*)/*F* and concentration of ferric ion was found. The calibration equation can be fitted into: *Y* = 0.6377*x* + 0.0113 (*R*^2^ = 0.996), where *F* and *F*_0_ represents the fluorescence intensity with and without the addition of ferric ions, respectively. According to the triple signal-to-noise ratio rule, the limit of detection was calculated to be 1.4 μM with a linear range from 1.4 μM to 0.8 mM, superior to the results from previous reports [35–37]. Note that the deviation in the concertation from 1.0 to 1.5 mM can be attributed to the limited concentration of MQDs. The detailed comparison of sensing performance between present MQDs and previous materials is shown in Table 1. Herein, it is worthy to be mentioned that the standard of iron content in drink water (regulated by the WHO) and blood is 5.36 μM and 20–29 μM, respectively, which can be achieved by the present MQDs-based sensor.

**Table 1:** Comparison of sensing performance between present MQDs and previous materials

Sensing materials	Sensitivity	Detection limit (μM)	References
Carbon dots from a-CD	–	6.5	Rasoulzadeh et al. [38]
N,P-co-doped CQDs	–	20	Sahu et al. [39]
CQDs from L-glutamic acid	–	4.67	Shi et al. [40]
Carbazole-based Schiff-base	8.33 × 10^4^ M^−1^	3.02	Li et al. [41]
PHBS sensor	2 × 10^6^ M^−1^	170	Sayed et al. [42]
DMSO-MQDs	–	0.31	Liu et al. [13]
Present MQDs	0.6377 mM^−1^	1.4	This work

**Fig. 5:**
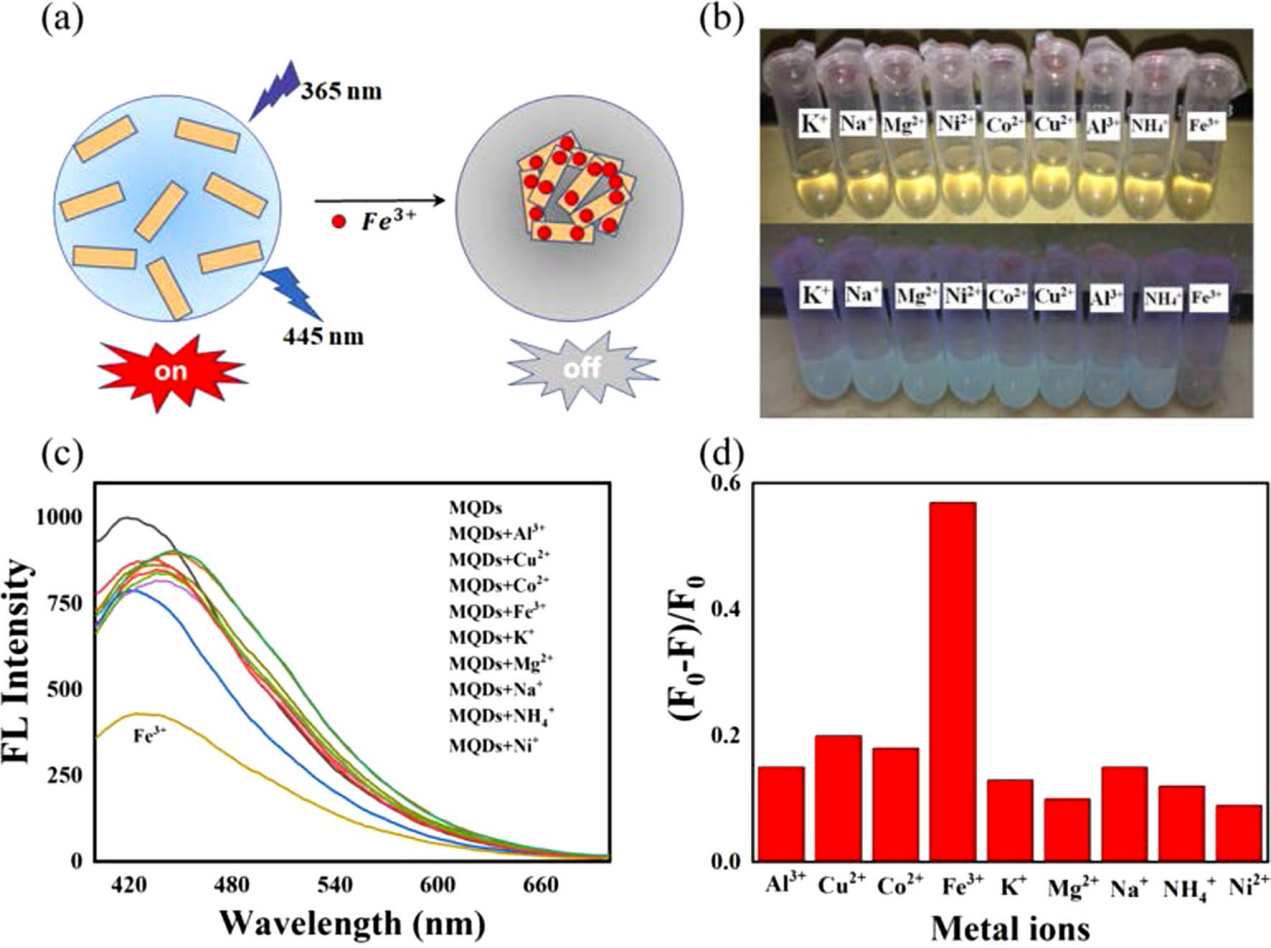
**a** Schematic diagram for the fluorescence quenching mechanism of MQDs by ferric ions; **b** the picture for the MQDs solutions with the addition of different metal ions (0.8 mM) under the visible and ultraviolet light; **c** the fluorescent curves of MQDs solution with the different metal ions (0.8 mM); and **d** the fluorescent intensity change of MQDs toward different metal ions collected from the curves in (**c**)

To investigate the potential quenching mechanism, zeta potential and UV–Vis spectra experiments were conducted. As shown in Fig. 4c, the zeta potential of − 10.9 mV was determined for the prepared MQDs. Based on the positive charge of metal ions, this indicates that a strong electrostatic interaction possibly occurs between metal ions and quantum dots. The Fe^3+^ with the higher positive charge and strong oxidation ability not only induced a stronger interaction, but also caused the subsequent REDOX reaction, which may play key roles in the fluorescence quenching of MQDs [43]. As a comparison, the Al^3+^ cannot effectively quench the fluorescence of MQDs resulted from the loss of oxidation capacity. In addition, the ferrous ions can also cause the decrease in fluorescent intensity, which may be the strong coordination interaction between iron and nitrogen. Furthermore, in Fig. 4d, UV–Vis spectra showed a pronounced decrease in the absorption intensity of the supernatant after the addition of iron, when compared to the pristine solution. Meanwhile, in inset of Fig. 4d the digital electronic images visualized the conspicuous precipitation. From this, it can be concluded that the iron ions induced the aggregation of MXene quantum dots through the electrostatic interaction, REDOX reaction, and coordination interaction, leading to the final fluorescence quenching (Fig. 5a).

Selectivity is another important factor for evaluating the performance of sensors. Herein, to show the specificity of the present sensor, the fluorescence intensity changes were investigated in the presence of different interferences, including the metal ions of K^+^, Na^+^, Mg^2+^, Ni^2+^, Co^2+^, Cu^2+^, Al^3+^, and NH_4_^+^. As shown in Fig. 5b, the MQDs solution containing other metal ions displayed the same light yellow under the daylight, while the fluorescence quenching was observed from the mixtures with the ferric ions under ultraviolet light. In Fig. 5c, the negligible fluorescence intensity fluctuations were found after the addition of interference metal ions (0.8 mM), compared to that caused by the ferric ions with same concentration. Furthermore, Fig. 5c exhibits the peak value changes of fluorescence intensity from the mixtures with the same concentration of different metal ions. Compared to that of other ions, the peak values of ferric ions obviously changed, suggesting that the prepared luminous MXene quantum dots will be a promising selective probe for the detection of ferric ions.

Herein, based on the above sensitivity and selectivity, the potential application of present MQDs for sensing of Fe^3+^ in the tap water was also achieved. Despite of the various impurities such as minerals and organics, the present MQDs are still sensitive to iron ions. With the addition of 0.7 mM Fe^3+^, the recovery from three independent replicates was 104.57%, 103.25%, and 97.9%, respectively, as shown in Table 2. This suggests the promising application of the MQDs in detecting Fe^3+^ in real environmental sample. We believe that a portable sensor for Fe^3+^ will be constructed in the near future, when combing with the integrated circuits and electronic chips.

**Table 2:** The results from three independent experiments when the Fe^3+^ with the concentration of 0.7 mM was added into the tap water

Test number	Fluorescent intensity (F)	Experimental results (mM)	Recovery (%)
1	453.62	0.73	104.57
2	458.12	0.72	103.25
3	478.86	0.69	98.0

## Conclusions

In summary, the MQDs with the blue fluorescence were synthesized through the facile intermittent ultrasound process in the presence of DMF solvent. Based on the electrostatic interaction between surface functional groups of quantum dots and iron ions, the sensitive and selective detection of Fe^3+^ was realized in this work. Meanwhile, the electrostatic-induced aggregation was also demonstrated. We believe that the obtained results will not only provide a new thought for synthesis of the MQDs, but also broaden the application areas.

## Data Availability

The data and conclusions in this work are all shown in this paper.

## Abbreviations

MQDs: MXene quantum dots
DMF: *N,N*-Dimethyl formamide
2D: Two-dimensional FTIR: Fourier-transform infrared spectroscopy
XPS: X-ray photoelectron spectroscopy
TEM: Transmission electron microscopy
